# Organic topological insulators (OTI): a dream coming true?

**DOI:** 10.1093/nsr/nwaa049

**Published:** 2020-03-28

**Authors:** Danfeng Zhi, Chong-an Di, Daoben Zhu

**Affiliations:** Beijing National Laboratory for Molecular Sciences, CAS Key Laboratory of Organic Solids, Institute of Chemistry, Chinese Academy of Sciences, China; University of Chinese Academy of Sciences, China; Beijing National Laboratory for Molecular Sciences, CAS Key Laboratory of Organic Solids, Institute of Chemistry, Chinese Academy of Sciences, China; University of Chinese Academy of Sciences, China; Beijing National Laboratory for Molecular Sciences, CAS Key Laboratory of Organic Solids, Institute of Chemistry, Chinese Academy of Sciences, China; University of Chinese Academy of Sciences, China

## Abstract

Experimental discovery of organic topological insulators (OTI) is a dream for both topological matters and organic materials. Despite great challenges, we anticipate that the dream will become a reality by engineered studies on materials chemistry, characterization techniques and device physics of conjugated molecules.

Topological insulators (TIs) are a class of materials with insulating bulk states but exotic metallic states on their surfaces [1]. Benefiting from topological boundary states, TIs have become one of the most important topics in condensed materials, not only because they offer a unique perspective for revealing the origin of various quantum phases, but also because of their potential applications in spintronics and quantum computing devices. The first experimental report of a two-dimensional (2D) TI in 2007 was based on observation of a quantum spin Hall insulator state in HgTe quantum [2]. Subsequently, several 2D and three-dimensional (3D) TIs, including HgTe, Bi_2_Te_3_, TlBiTe_2_ and Heusler compounds [3–7], were reported. Despite such achievements, chemists and physicists have sustained interest in exploring novel TIs. Organic TIs (OTIs), with their relatively weakly correlated nature, represent a dream category for researchers because of the potential unveiled topological behaviors of such TIs.

During recent decades, many inorganic materials have successfully been matched with their organic counterparts to enable various cutting-edge research frontiers. The OTIs offer a route to discovery of novel organic states and a move into a new research area. Organic materials are widely recognized by their abundant electronic and condensed structures *via* fine-tuned molecular engineering, which is critical to meet the requirements of TIs. However, their relatively weak interactions pose a great challenge to obtaining highly ordered materials for identification of OTIs. There is a knowledge gap between most existing organic materials and OTIs, and this raises the question: how can we search for or design an OTI?

## OTI: AN EMERGING FIELD AT THE THEORETICAL STAGE

**Figure 1.**
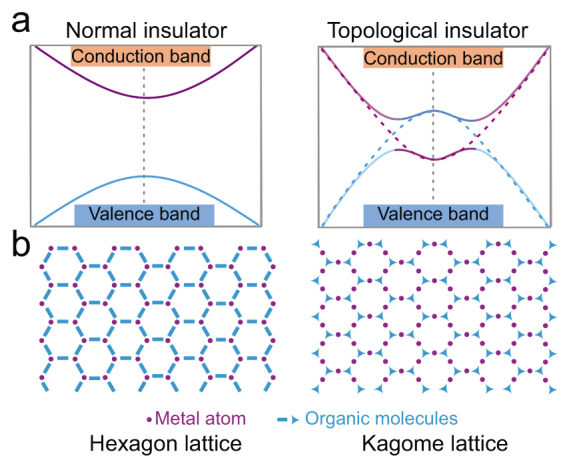
(a) Energy level of conventional insulator and topological insulator and (b) theoretical calculated lattices for organic topological insulators.

TIs are distinguished from conventional insulators by nontrivial topological invariants related to their bulk electronic structures (Fig. 1a) [7]. Notably, their electronic structures are further characterized by spin-orbit coupling and an odd number of Dirac-like edge states for 2D TI (or surface states for a 3D TI) connecting the conduction and valence edge at k-points. To fulfill these requirements, the desired OTI should possess strong spin-orbit coupling and well-defined lattice symmetry to ensure band inversion. This implies that heavy-element and small-bandgap semiconductors with hexagonal and Kagome lattices are promising TI candidates (Fig. 1b).

The existence of 2D OTI with hexagonal organometallic lattice was initially predicted by Liu *et al.* in 2013 [8]. The proposed molecule consists of a Pb atom bonded with three benzene rings to enable 3-fold rotational symmetry. Their simulation not only indicated an ultra-small bandgap of 8.6 meV open at k-point, but also demonstrated the nontrivial topological gapless edge stages that connect the band state forming a 1D Dirac cone. Thereafter, they predicted another OTI based on an experimental sample of a 2D organometallic framework, namely, Ni_3_C_12_S_12_ [9]. Unlike the aforementioned lattice, this sample possessed a Kagome lattice in which the metal was bonded with two neighboring organic molecular groups. However, its Fermi level should be fine-tuned by doping to shift into the Dirac-cone gap [8,10]. More interestingly, the same group of researchers proposed the existence of magnetic OTIs and flat-band based OTIs using triphenylmanganese and indium-phenylene organometallic framework as the model systems, respectively [11,12]. Inspired by these achievements, several theoretical studies have been performed to predict more OTI-based organometallic frameworks. In spite of this theoretical progress, OTIs have not yet been experimentally reported, even with the rapid developments of ordered organometallic frameworks. A possible reason might be the strong interactions of surface-synthesized materials with the substrates and/or insufficient quality of obtained samples to meet the critical requirement of OTI characterization.

## WHEN OTIs MEET ORGANIC THERMOELECTRIC MATERIALS

From an empirical point of view, many TI compounds, including Bi_2_Te_3_, Sb_2_Te_3_, *etc.*, are also excellent thermoelectric (TE) candidates as they share similar features in terms of heavy elements and narrow band gaps [13]. These common characteristics imply an underlying mechanism between TI boundary states and TE conversion. For TE materials, the performance is determined by the dimensionless figure of merit ZT = S^2^σT/κ, where S, σ, T and κ are the Seebeck coefficient, electrical conductivity, absolute temperature and thermal conductivity, respectively. It is therefore natural to expect that the TI boundary states contribute to improved S and σ, but decreased κ, separately or synergistically, in a unique manner. Following this concept, many theoretical and experimental studies have been conducted to understand the physical insight. A general conclusion is that topologically protected boundary states work as superior conducting channels for electronic transport, while the induced backscattering dominates phonon transport. The decoupled transport is thus consistent with the ‘phonon-glass, electron-crystal’ concept and is responsible for the significantly modulated TE performance in TIs.

In contrast to many inorganic TE, most organic TE (OTE) materials do not show strong spin-orbit coupling because of the absence of heavy elements. This makes the incorporation of OTI and OTE particularly challenging. Thanks to the reports on metal-organic coordination polymers such as Poly(Ni-ett) and doped materials with involved heavy elements [14,15], we believe that OTE candidates hold promise to exhibit TI behaviors and might exhibit unprecedented TE performance endowed by the TI boundary states.

## CHALLENGES TOWARDS A BRIGHT FUTURE

Although scientists have predicted the existence of OTIs *via* theoretical calculation, the experimental trials have not yet been successful and face many fundamental challenges. Do the predicted materials truly possess TI behavior? How can the OTI materials be identified with experimental methods? Where is the starting point to manipulate the properties of OTIs? So far, these open questions remain unanswered and may do so for a long time.

Engineered studies in the following aspects are essential to facilitate the realization of OTIs. Firstly, the synthesis and growth of ultra-high quality, large-area single crystals based on predicted molecules and other well-designed metal-organic coordination materials could provide a shortcut to accelerate the screening process of OTIs. Secondly, a precisely modulated Fermi level and bandgap are key for OTIs, thus requiring chemically tailored molecules especially with fine-tuned heavy elements. Thirdly, the experimental confirmation of TIs relies on advanced spectroscopy techniques, which makes high-resolution angle-resolved photoemission spectroscopy (ARPES) and scanning transmission electron micro-scopy (STEM) measurement of organic materials of vital importance. Fourthly, modulation of spin orbital coupling with magnetic and/or electric fields deserves attention to accelerate the discovery of novel OTIs. Last but not least, combined studies on OTI and OTE materials offer a powerful strategy to develop state-of-the-art organic materials with multifunctional properties. Breakthroughs in these aspects could contribute to development of OTIs in an unexpected manner.

Based on the aforementioned theoretical achievements and challenges, it is clear that great opportunities lie ahead in this unexplored field. Future progress in OTIs will not only advance the understanding of TI boundary states of condensed matters, but also offer new insights into the physical properties of organic materials towards their quantum world. We expect that this ambitious dream will become a reality in the near future.
